# Characterization of Ocular Developmental Disorders in the Israeli Population: Genotype–Phenotype Correlations and Novel Candidate Genes

**DOI:** 10.3390/biom16081219

**Published:** 2026-08-21

**Authors:** Yakov Rabinovich, Yoav Vardizer, Shirley Pincovich, Marva Wolowelsky, Sofia Kulyamzin, Miriam Ehrenberg, Shiri Zayit-Soudry, Inbal Man Peles, Rina Leibu, Nitza Goldenberg-Cohen, Tamar Ben-Yosef

**Affiliations:** 1 Department of Ophthalmology, Bnai Zion Medical Center, Haifa 3339419, Israel; rabinovy@mcmaster.ca (Y.R.); yoavardizer@gmail.com (Y.V.); shirleyp@gmc.gov.il (S.P.); inbal.man-peles@b-zion.org.il (I.M.P.); 2 The Ruth and Bruce Rappaport Faculty of Medicine, Technion-Israel Institute of Technology, Haifa 3109601, Israel; marva.w@campus.technion.ac.il (M.W.); sofia.kuly@campus.technion.ac.il (S.K.); 3 Department of Ophthalmology, Schneider Children’s Medical Center of Israel, Petah Tikva 4920235, Israel; mirieh1@clalit.org.il; 4 Gray Faculty of Medical and Health Sciences, Tel Aviv University, Tel Aviv 6139001, Israel; shirisou@clalit.org.il; 5 Department of Ophthalmology, Rabin Medical Center, Petah Tikva 4941492, Israel; 6 Department of Ophthalmology, Rambam Health Care Campus, Haifa 3109601, Israel; rinaleibu@gmail.com

**Keywords:** microphthalmia, anophthalmia, coloboma, genetics, eye

## Abstract

Microphthalmia, anophthalmia and ocular coloboma (MAC) are rare developmental eye disorders. Although over 100 causative genes have been identified, the molecular spectrum and genotype–phenotype correlations remain incompletely understood, particularly in genetically diverse populations. We set out to molecularly characterize MAC in the Israeli population. Forty-seven MAC-affected individuals from 43 unrelated families were enrolled. DNA of all probands was subjected to whole exome sequencing. The most common phenotype was microphthalmia (64% of patients). Definite or possible molecular diagnoses were achieved in 13/43 probands (30%) and involved 10 different genes (*MFRP*, *SMO*, *GJA8*, *SOX2*, *RARB*, *TSPAN12*, *SHH*, *PTPN11*, *BEST1*, and *TP63*). An in vitro splicing assay was used to explore the pathogenicity of a variant in the *SMO* gene. Following stringent filtering of exome data, 226 rare possibly pathogenic variants were identified in 218 genes not previously associated with MAC. The rate of molecular diagnosis achieved in this Israeli MAC cohort is similar to the reported range in other studies. The results further demonstrate the genetic heterogeneity of MAC, while supporting the involvement of complex inheritance and/or environmental factors in many of the cases. Further studies are required to reveal these underlying etiological factors, and to support the novel genotype–phenotype associations suggested here.

## 1. Introduction

Abnormalities of early ocular development exist along a spectrum collectively referred to as microphthalmia, anophthalmia, and coloboma (MAC). At the severe end of this continuum lies true anophthalmia, characterized by the complete absence of ocular tissue. Clinical anophthalmia describes the absence of a visible globe on examination, despite the possible presence of rudimentary ocular tissue on imaging or histologic evaluation. Coloboma represents a segmental defect resulting from incomplete closure of the embryonic fissure and may present with a wide range of severity, including subtle phenotypes. Microphthalmia, in contrast, is defined by a formed globe that is reduced in size and may occur in isolation or in association with coloboma. Although MAC is rare, with a reported birth prevalence of approximately 3–12 per 100,000 [1,2,3,4], it carries profound implications for affected individuals and families, as children born with MAC often require lifelong multidisciplinary ocular rehabilitation and ongoing psychosocial support. In practice, these needs are not consistently addressed. Even in recent cohorts, only 60% of anophthalmic patients obtained an ocular prosthesis, with cost cited as the most common barrier [5].

MAC is clinically heterogeneous, differing in laterality and in the extent of associated ocular and systemic involvement. Importantly, a substantial proportion of patients have extra-ocular manifestations, with systemic involvement reported in roughly one-third to over half of cases across cohorts [4,6,7]. Complex ocular cases with additional ocular comorbidities are also common, reported in approximately 44–60% of individuals in selected series, with cataract and anterior segment abnormalities being the most common [3]. Given both the frequency and breadth of extra-ocular involvement, MAC patients require prompt structured systemic evaluation and multidisciplinary care, even when the ocular phenotype appears isolated.

The etiology of MAC is complex and includes Mendelian inheritance modes (autosomal recessive (AR), autosomal dominant (AD) and X-linked) and multifactorial inheritance, which involves both genetic and environmental risk factors. In addition to gene mutations, genetic causes also include chromosomal abnormalities, which account for 20–30% of cases. De novo mutations are frequently detected. Even in cases with Mendelian inheritance, non-penetrance and variable expressivity are commonly observed, suggesting the influence of genetic and/or environmental modifiers. To date, more than 90 genes have been associated with MAC [8]. These genes are involved in multiple aspects of eye development, including early eye development (*OTX2*, *RAX*, and *PAX6*), formation of the lens placode (*PAX6*, *SOX2*, and *BMP4*), optic cup morphogenesis (*ALDH1A3*), neural crest cell migration (*PAX6*, *SOX10*, *OTX2*, *RAX*, *BMP4*, and *FOXE3*), retinal development (*SOX2*, *PAX6*, *OTX2*, *VSX2*, *RAX*, *FOXE3*, *SHH*, and *MITF*), and more (reviewed in [8]). Nevertheless, not all cases have an identifiable genetic cause, and some may exclusively result from environmental and prenatal influences, such as gestational infectious diseases, nutritional deficiencies and exposure to teratogens [8].

With modern sequencing-based testing, the reported molecular diagnostic yield is variable, reaching 18–60% across published cohorts [6,9,10,11,12,13]. In a prospective cohort of MAC patients referred to Moorfields Eye Hospital, molecular testing using targeted gene panels, whole exome sequencing (WES), whole genome sequencing (WGS), and microarray comparative genomic hybridization (CGH) achieved a modest overall molecular diagnostic rate of 33% [6]. An even lower molecular diagnostic rate of 18% was achieved in a cohort of 100 MAC probands analyzed by WGS as part of the French Genomic Medicine Initiative [9].

Israel is uniquely well-suited for population-based investigation of genetic conditions, including MAC, due to its complex demographic diversity, the presence of multiple founder effects and genetic isolates, and the opportunity to study both consanguineous and outbred groups within the same healthcare framework [14,15]. Nonetheless, integrated MAC datasets combining deep phenotyping with systematic molecular testing in Israel remain limited. In this study, we performed comprehensive clinical phenotyping and exome analysis of individuals with MAC in Israel to define the clinical spectrum, determine diagnostic yield, characterize the underlying molecular architecture, and assess genotype–phenotype relationships relevant to clinical care and genetic counseling.

## 2. Materials and Methods

### 2.1. Study Participants

The study was conducted in accordance with the Declaration of Helsinki and was approved by the local Institutional ethics committees at Bnai Zion Medical Center, Schneider Children’s Medical Center of Israel and Rambam Health Care Campus. Written informed consent was obtained from all participants or legal guardians. Additional family members were recruited when available. Phenotypic data were collected at enrollment from standardized ocular examination and review of prior clinical records. Extra-ocular features were obtained from medical history and relevant specialist evaluations.

### 2.2. Genetic Analyses

Genomic DNA was obtained from peripheral blood and/or buccal swab samples using standard protocols. WES was performed at the Center for Genomic Sequencing at Tel Aviv Sourasky Medical Center, using the Nextera DNA Flex Pre-Enrichment Library Prep (Illumina, San Diego, CA, USA) and the xGen Exome Hyb Panel & xGen Human Mitochondrial DNA Hyb Panel (Integrated DNA Technologies, Coralville, IA, USA). Sequence reads were aligned to the human reference genome (GRCh37/hg19) (http://genome.ucsc.edu/ (accessed on 16 August 2026)). Average variant depth was 80X and percent of target covered (depth ≥ 20) was 95% (refseq genes). Variant annotation, filtering, and prioritization were conducted using the Franklin by Qiagen web-based platform (https://franklin.genoox.com/clinical-db/home (accessed on 16 August 2026)). High-quality variants were filtered based on the following criteria: (1) allele frequency (AF) ≤1% in the Genome Aggregation Database (gnomAD) v4.1.0; (2) variants located in exons or in splice regions (±10); and (3) variants with predicted deleterious or uncertain effects (including stop gain, stop loss, start gain, start loss, deletions/insertions, missense, and candidate splice-affecting variants). Variants remaining after steps 1–3 were first filtered for location within known MAC genes. Then, all variants remaining after steps 1–3 were filtered again, based on the following criteria: (4) location within genes which lack a known associated phenotype; (5) location within genes which are expressed in the eye; and (6) variants reported ≤20 times by the Franklin community. The filtering strategy is demonstrated in Figure S1. Sanger sequencing was used for confirmation of putative disease-causing variants and for segregation analysis.

### 2.3. Bioinformatics

The pathogenicity of missense variants was evaluated based on the Franklin aggregated prediction score, which is based on scores obtained by multiple prediction tools, including: REVEL [16], MutationAssessor/r3 [17], SIFT (https://sift.bii.a-star.edu.sg/ (accessed on 16 August 2026)), Polyphen-2 (https://genetics.bwh.harvard.edu/pph2/ (accessed on 16 August 2026)), MutationTaster (https://www.mutationtaster.org/ (accessed on 16 August 2026)), FATHMM (v2.3) [18], DANN [19], MetaLR [20], PrimateAI (https://primateai3d.basespace.illumina.com/ (accessed on 16 August 2026)) and BayesDel [21]. The putative effect of certain variants on splicing was evaluated based on the following prediction tools: SpliceAI (v1.3.1) [22], dbscSNV_AdaBoost [23] and dbscSNV_RandomForest [24]. Analysis of copy number variants (CNVs) was performed by Franklin’s advanced AI algorithm “Rainbow” [25]. Gene-associated phenotypes were determined based on OMIM—Online Mendelian Inheritance in Man (https://omim.org/ (accessed on 16 August 2026)). Expression in the eye was determined based on the EyeBrowse track hub on the University of California at Santa Cruz Genome Browser (https://genome.ucsc.edu/ (accessed on 16 August 2026)).

### 2.4. Minigene Splice Assay

To generate wild-type (WT) and variant minigene constructs, DNA segments incorporating exons 5, 6 and 7 of the *SMO* gene, each flanked by 157–333 bp of intronic sequences, were PCR-amplified from genomic DNA of patient NGC12-002. PCR products were sub-cloned into the pGem-T Easy vector (Promega, Madison, WI, USA) and sequenced. Fragments were then cloned in tandem into the pCMV-Script mammalian expression vector (Stratagene, Agilent Technologies, Santa Clara, CA, USA). Two constructs were generated, with either a G or a C present at position +3 of intron 6. HeLa cells were transfected with the constructs using jetPEI reagent (Polyplus-transfection, Strasbourg, France) and cultured in DMEM supplemented with 10% fetal bovine serum, 1% penicillin/streptomycin, and 1% glutamine (Biological Industries, Beit HaEmek, Israel) at 37 °C with 5% CO_2_. After 24 h, total RNA was isolated using TRI reagent (Sigma-Aldrich, St. Louis, MO, USA), and 1 μg of RNA was reverse-transcribed in a 20 μL reaction using UltraScript cDNA Synthesis kit (PCR Biosystem Ltd., London, UK). In total, 2 μL of the resulting cDNA were amplified by PCR using a forward primer located in exon 5 and a reverse primer located in exon 7. PCR products were subjected to electrophoresis on a 2% agarose gel. Relative levels of RT-PCR products were quantified with the TotalLab software (version 13.1, Newcastle upon Tyne, UK). Products of various sizes were gel-purified using the Wizard SV gel and PCR Clean-Up kit (Promega) and sub-cloned into the pGem-T Easy vector. Multiple independent clones of each size range were sequenced.

## 3. Results

### 3.1. Demographic and Clinical Characteristics of the MAC Cohort

**Figure 1:**
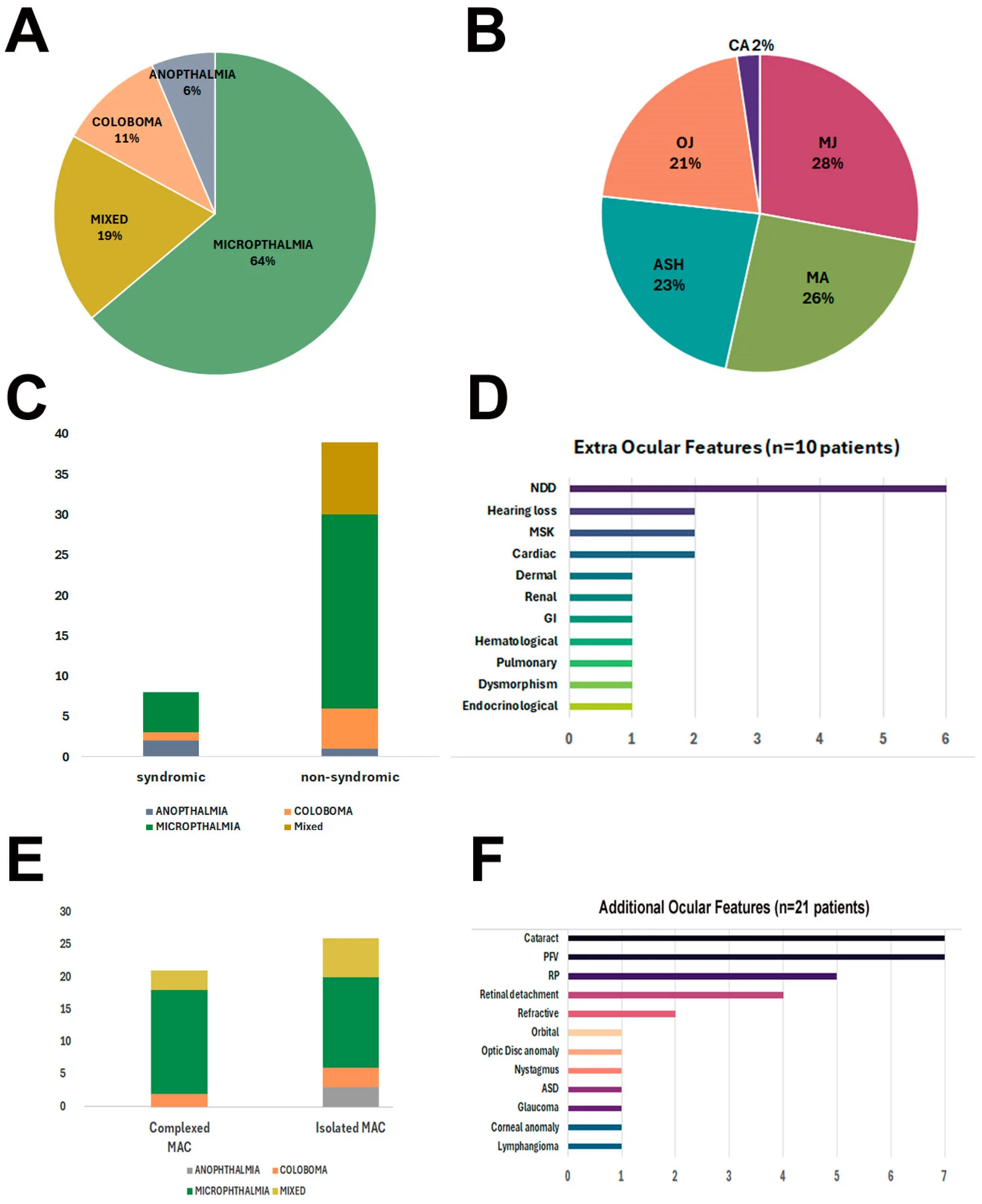
Demographic and clinical characteristics of the MAC cohort (*n* = 47 patients from 43 families). (**A**) Phenotypic distribution of recruited patients. (**B**) Ethnic distribution of recruited families. ASH, Ashkenazi Jewish; CA, Christian Arab; MA, Muslim Arab; MJ, mixed Jewish; and OJ, Oriental Jewish. (**C**) Syndromic versus non-syndromic presentation stratified by MAC phenotype. (**D**) Distribution of extra-ocular features. Categories are shown as the number of patients with at least one feature in each system. GI, gastrointestinal; MSK, musculoskeletal; and NDD, neurodevelopmental disorder. (**E**) Isolated MAC versus complex MAC stratified by phenotype. Isolated MAC denotes MAC without additional ocular abnormalities; complex MAC denotes MAC with additional ocular findings. (**F**) Distribution of additional ocular findings among complex MAC patients. Categories are shown as the number of patients with at least one additional ocular finding. ASD, anterior segment dysgenesis; PFV, persistent fetal vasculature; and RP, retinitis pigmentosa.

A total of 47 affected individuals (20 males and 27 females) from 43 families with MAC spectrum were recruited (Table S1). Median age at recruitment was five years old (IQR 2–27; range 0.3–73). The primary phenotype was microphthalmia in 30/47 patients (64%), followed by a mixed phenotype (involving more than one MAC feature) in 9/47 (19%), coloboma in 5/47 (11%), and anophthalmia in 3/47 (6%) (Figure 1A). Participating families were of diverse origins, including mixed Jewish (*n* = 12), Muslim Arabs (*n* = 11), Ashkenazi Jewish (*n* = 10), Oriental Jewish (*n* = 9), and Christian Arab (*n* = 1) (Figure 1B). Laterality information indicated predominantly unilateral involvement (*n* = 30) compared with bilateral disease (*n* = 17). Ten patients (21%) had extra-ocular features (Figure 1C,D), and 21 (45%) had additional ocular findings beyond the core MAC phenotype (Figure 1E,F). Microphthalmia was the most common phenotype regardless of extra-ocular or additional ocular features (Figure 1C,E).

### 3.2. Genetic Findings in MAC Families

DNA of all probands was subjected to WES. In addition to variant annotation, WES data was also analyzed for CNVs. Variant filtering strategy aimed to identify high-quality rare variants, located in exons or in splice regions, with predicted deleterious or uncertain effects. In the first stage, we focused on variants located within genes with a known association to MAC (Figure S1).

**Figure 2:**
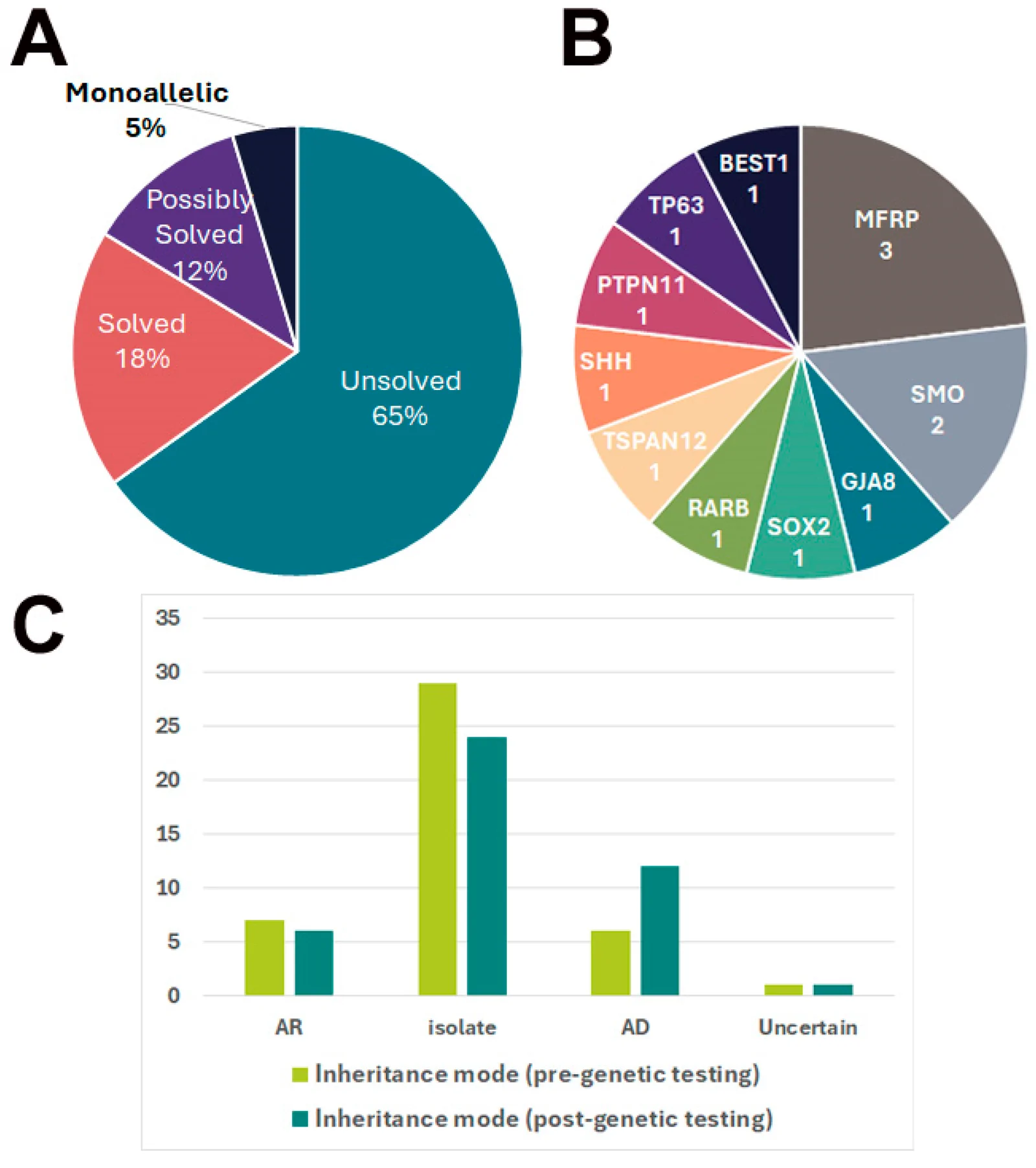
Genotypic characteristics of the MAC cohort. (**A**) Genetic diagnosis status of the cohort following genetic analysis, categorized as solved, possibly solved, monoallelic, or unsolved. (**B**) Genes in which pathogenic and possibly pathogenic variants were identified among solved and possibly solved families, demonstrating marked genetic heterogeneity. The numbers below each gene name correspond to the number of families in which pathogenic variants in this gene were detected. (**C**) Classification of inferred inheritance mode before and after genetic testing. AD, autosomal dominant; AR, autosomal recessive.

A definitive genetic diagnosis was established in eight families, and five families were considered possibly solved (Table A1 and Figure 2A). The rate of genetically diagnosed/possibly diagnosed families in this cohort is therefore 30%. In two additional families, heterozygous suspected variants were identified in MAC-associated genes known to be inherited as AR (Table A1). Patients from these families might have a second pathogenic allele which was missed, or, alternatively, they may be coincidental carriers for the observed variants, while the underlying cause for their condition lies elsewhere. In either case, given that no second pathogenic allele was detected, these families were categorized as monoallelic and were considered genetically unsolved.

Pedigree-based inheritance assignments were concordant with genetic-based inference in 6/13 (46%) of solved and possibly solved families. Notably, 5/29 (17%) of families originally classified as isolated cases based on pedigree were reclassified to Mendelian inheritance (AR/AD) (Figure 2C and Table S1).

Overall, pathogenic and likely pathogenic variants were identified in 10 different genes (Table A1 and Figure 2B). *MFRP* was the most common causative gene (30% of solved/possibly solved families and 7% of all families). Homozygous pathogenic variants in this gene were observed in four patients from three consanguineous families, with a phenotype involving both microphthalmia/nanophthalmos and retinal dystrophy (families TB1517, TB1110, and Tb533/621). This phenotypic combination is characteristic for individuals with bi-allelic pathogenic variants in the *MFRP* gene [26,27]. Another recessively inherited variant was detected in patient NGC34-001. This patient, affected with a combination of microphthalmia, retinal detachment and persistent fetal vasculature (PFV) (as well as developmental delay), was homozygous for a likely pathogenic variant in the *TSPAN12* gene (c.542G>T; p.(Cys181Phe)). Pathogenic variants in this gene are associated with familial exudative vitreoretinopathy, a disorder characterized by defects in the development of retinal vasculature with either an AD or AR inheritance mode [28]. Patient NGC34-001 belongs to an extended consanguineous Muslim Arab family from Northern Israel. The specific variant identified in this patient was previously reported in additional members of this extended family, in which it segregated in an AR mode. Affected individuals presented with abnormal vitreoretinal vasculature, retinal detachment and microphthalmia [29].

Nine heterozygous variants were observed in genes associated with AD conditions (*GJA8*, *SOX2*, *RARB*, *SMO*, *SHH*, *PTPN11*, *BEST1*, and *TP63*) (Table A1). DNA of additional family members was available for segregation analysis for eight of these variants (Table S2). This revealed two cases in which the variant was inherited from an affected parent (*SMO* and *TP63*) and two de novo events, for which both parents were confirmed to be WT (*RARB* and *PTPN11*). Four variants were inherited from supposably unaffected parents, suggesting incomplete penetrance or variable expressivity (Table S2). One of these cases is proband NGC14-001, who has microphthalmia, coloboma and microcornea. She is heterozygous for a rare variant in *BEST1*: c.1144G>A;p.(Glu382Lys). This variant, defined as a variant of unknown significance (VUS), was inherited from her unaffected father (Table S2). Nevertheless, her paternal grandmother (not recruited for this study) is also affected by microphthalmia, and her parents had a prior aborted pregnancy due to fetal ocular tissue maldevelopment, further supporting an AD inheritance with incomplete penetrance.

In family TB1340, the proband presented with coloboma and cataract. CNV analysis of WES results revealed a heterozygous deletion of 0.8 Mb on chromosome 1q21.1-q21.2. This is a recurrent deletion reported in multiple individuals worldwide [30]. The deletion encompasses 32 genes (Table S3), only two of which have a confirmed association with known Mendelian phenotypes in humans: *GJA8* and *GJA5*. *GJA8*-associated phenotypes range from isolated cataracts to a combination of ocular developmental abnormalities, including microphthalmia and cataracts with/without sclerocornea/microcornea, with an AD inheritance mode, which is in agreement with the phenotype observed in our patient [31,32]. Heterozygous missense variants in *GJA5* have been associated with AD atrial fibrillation. These variants most probably lead to a gain-of-function [33], while the deletion in our patient leads to a loss-of-function, therefore explaining the lack of a cardiac phenotype in this patient.

Syndromic MAC forms were identified in two solved families. Proband NGC9-001, affected with microphthalmia as well as dysmorphism, neurologic, cardiac, pulmonary, skeletal and diaphragm abnormalities, carried a de novo heterozygous *RARB* variant consistent with microphthalmia, syndromic 12 [34]. Proband NGC10-001 presented with anophthalmia, developmental delay and hypopigmented streaky skin lesions. She harbored an ultra-rare heterozygous variant in *SMO*. Heterozygous variants in this gene are associated with Curry–Jones syndrome, a multisystem disorder characterized by patchy skin lesions, iris colobomas, microphthalmia and cognitive impairment, among other features [35].

### 3.3. New or Rare Genotype–Phenotype Correlations

Five families were defined as possibly solved. In two of them, rare variants with pathogenic or uncertain predictions were found in genes for which an association with MAC is very rare and not definite. Proband NGC4-001 has microphthalmia and anterior segment dysgenesis. He was found to be heterozygous for a rare variant in *PTPN11*. This variant, c.794G>A; p.(Arg265Gln), that arose de novo (Table S2), is a recurrent mutation which was reported in multiple individuals with Noonan syndrome, an AD disorder characterized by short stature, facial dysmorphism, congenital heart defects and additional multisystemic abnormalities [36]. Pathogenic variants in *PTPN11* are associated with additional conditions, including LEOPARD syndrome. The common ophthalmic phenotypes associated with these conditions are eyelid and external eye abnormalities, as well as strabismus, refractive errors and amblyopia [37,38]. MAC is an uncommon finding in these patients. The combination of LEOPARD syndrome and anophthalmia was previously reported in one child with a *PTPN11* pathogenic variant [39]. The patient reported here does not meet diagnostic criteria for the Noonan or LEOPARD syndromes, and it is unclear whether the *PTPN11* variant underlies his microphthalmia.

Proband NGC18-001 has anophthalmia, polydactyly, oligodactyly, and a ventricular septal defect, as well as polycystic kidney disease. WES revealed a pathogenic *PKD2* variant, underlying polycystic kidney disease, and a *TP63* variant characterized as a VUS. *TP63*-associated conditions (including ADULT syndrome) include limb, hair, and teeth abnormalities, with cleft lip/palate being a prominent feature [40]. MAC is uncommon in these patients. The combination of ADULT syndrome with microphthalmia was previously reported in one child with a likely pathogenic variant in *TP63* [39]. The patient reported here presents with limb abnormalities, which might be consistent with ADULT syndrome, but whether the *TP63* variant underlies his anophthalmia as well is unclear.

### 3.4. Evaluation of a Splice-Region Variant in SMO by a Minigene Splice Assay

**Figure 3:**
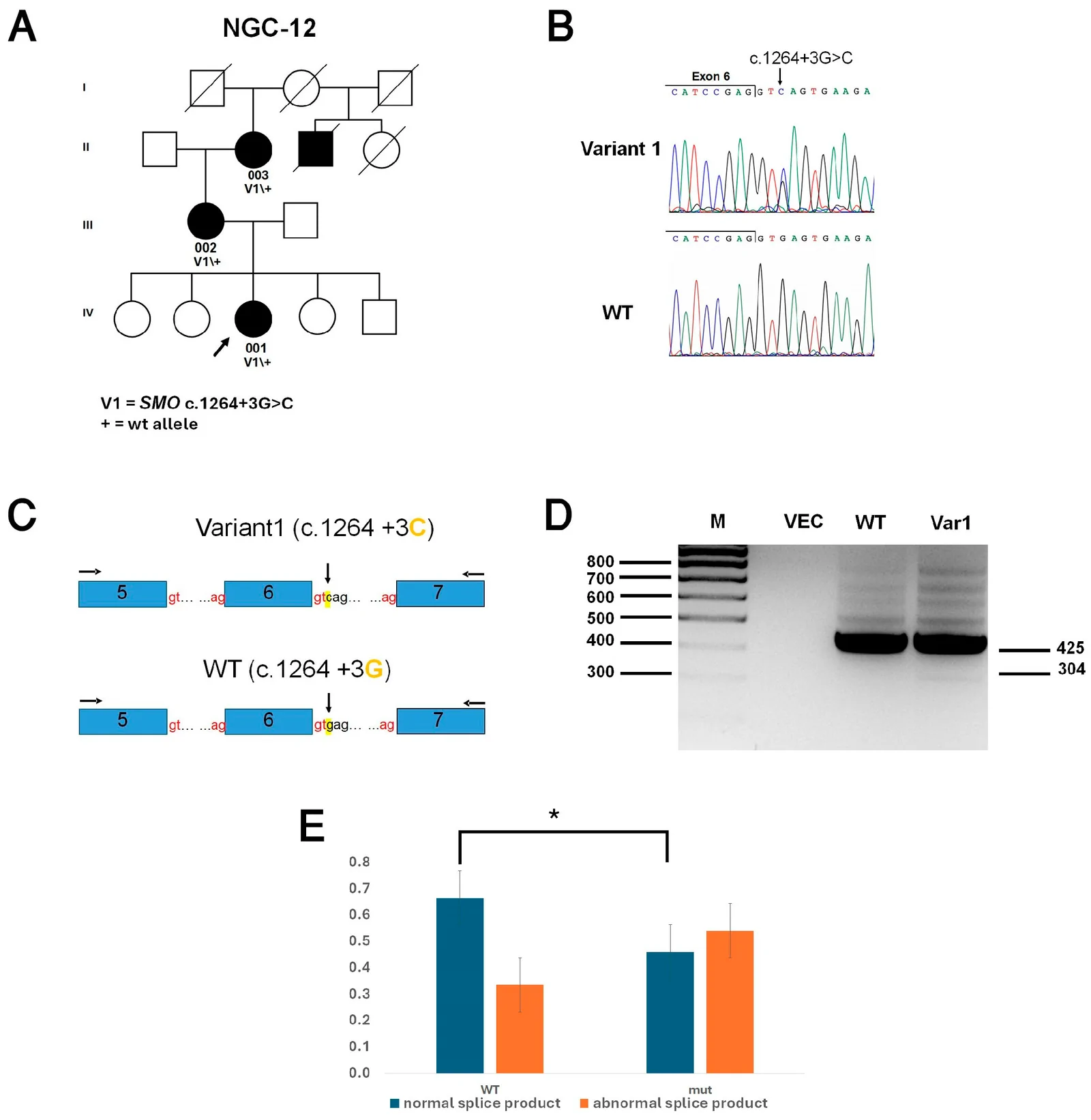
Identification and characterization of the *SMO* c.1264+3G>C variant. (**A**) Pedigree of family NGC12. Filled symbols denote affected individuals, whereas clear symbols indicate unaffected individuals. The proband is marked by an arrow. Genotypes of tested family members are indicated below them. (**B**) Nucleotide sequence traces of *SMO* exon-intron 6 boundary, showing wild-type (WT) and variant sequences. The c.1264+3G>C variant (indicated by an arrow) affects the +3 position of the intron 6 donor splice site. (**C**) Schematic representation of the minigene constructs containing *SMO* exons 5, 6, and 7 (blue boxes) and the intervening intronic regions. Horizontal arrows indicate the positions of primers used for RT-PCR analysis. Vertical arrows indicate the c.1264+3 position. (**D**) Agarose gel electrophoresis of RT-PCR products generated from WT and variant (Var1) constructs. M, size marker; VEC, empty vector control. (**E**) Quantitative comparison of normal and aberrant splicing products derived from the *SMO* c.1264+3G and c.1264+3C constructs. Blue bars represent the percentage of normal splice products, while orange bars represent the percentage of aberrant splice products. The experiment was conducted in *n* = 6 biological repeats. Statistical significance was assessed using a two-tailed Student’s *t*-test (* *p* < 0.05).

In family NGC12, the proband (NGC12-001), as well as her mother (NGC12-002) and maternal grandmother (NGC12-003), have microphthalmia, suggesting AD inheritance (Figure 3A). WES analysis of the mother identified an ultra-rare heterozygous variant in *SMO*, a gene associated with AD inheritance. This variant, c.1264+3G>C, classified as a VUS, was also found heterozygously in the proband and the maternal grandmother.

Given that the observed variant is located within the intron 6 splice region (Figure 3B), we hypothesized that it may disrupt normal splicing. To evaluate this potential pathogenic mechanism, we performed an in vitro minigene splicing assay. For this purpose, we created minigene constructs harboring *SMO* exons 5 to 7, flanked by 157-333 bp of intronic sequences, with either a G or a C at position +3 of intron 6 (Figure 3C). Constructs were transfected into HeLA cells, followed by RNA extraction and RT-PCR analysis. The WT construct (+3G) yielded a major 425 bp product and multiple minor larger products (500–800 bp). For the variant construct (+3C), the same major product was observed, and multiple minor larger products were also present, but these minor products exhibited significantly higher intensity. Furthermore, a distinct lower product of 301 bp was detected (Figure 3D). All products were subcloned, and Sanger sequencing was performed on multiple independent clones (see Supplementary Material). The results showed that the major product represented the normal splicing isoform, with correct removal of introns 5 and 6. The additional higher bands arose from aberrant splicing via cryptic splice site usage, whereas the distinct lower band resulted from complete exon 6 skipping. Band quantification revealed a statistically significant shift in the relative proportions of splice isoforms between the samples. The WT construct resulted in 70% normal products and 30% aberrant products. In contrast, the variant construct resulted in 46% normal products, and 54% aberrant products (Figure 3E). These results indicate that c.1264+3G>C is a hypomorph, which reduces the splicing efficiency of *SMO* intron 6. While evaluation of this variant was performed in vitro, and may not fully recapitulate the retinal microenvironment, given its rarity (not present in gnomAD) and its co-segregation with microphthalmia across three consecutive generations, it might be responsible for the phenotype in this family.

### 3.5. Genetic Findings Associated with Non-MAC Phenotypes

As indicated before, ten of the MAC patients had extra-ocular features. Genetic analysis revealed that in three of them (families NGC9, NGC10 and NGC18) these features were probably part of a syndrome, while in five of them genetic variants were identified that underlie extra-ocular features, but not MAC, indicating co-occurrence of distinct genetic conditions (Table A1). These include polycystic kidney disease (family NGC18, *PKD2* variant) [41], possible autoimmune disease related to JAK1 activation (family NGC11) [42], hereditary hearing loss (families ZKA1 and NGC25; *COL4A5* and *MPZL2* variants, respectively) [43,44] and a neuropsychiatric disorder (family NGC29, *ADGRL1* variant) [45].

### 3.6. Identification of Novel MAC Candidate Genes

As part of the second stage of the analysis, exomes of both solved and unsolved probands were subjected to an expanded search for additional rare variants, located within genes with no phenotypic association and expressed in the human eye (Figure S1). In total, 226 variants in 218 genes were identified (Table S4). We focused on genes in which suspected variants were detected in more than one patient. This led us to the identification of two main candidate genes: *MCFL2* and *HK3*.

*MCFL2* (NM_015078.4): Proband NGC3-001 (an isolate case of non-syndromic unilateral microphthalmia) was heterozygous for a missense variant, c.2207G>A; p.(Gly736Glu). This variant is located in the penultimate position of exon 19 and predicted to affect splicing (aggregated prediction score = 0.78, deleterious). Proband NGC6-001 (an isolate case of non-syndromic unilateral microphthalmia and coloboma) was heterozygous for a nonsense variant, c.2196C>A; p.(Cys732*). Both suspected variants in this gene are ultra-rare (not present in gnomAD). *MCF2L2* (KIAA0861) encodes for MCF.2 cell-line derived transforming sequence-like 2, a Rho-family guanine-nucleotide exchange factor [46]. It is not associated with a known Mendelian phenotype in humans.

*HK3*: Proband NGC17-001 (an isolate case of non-syndromic unilateral microphthalmia and coloboma) was heterozygous for a missense variant, c.1172T>C; p.(Val391Ala). This variant, defined as a VUS, is predicted to be deleterious (aggregated prediction score = 0.84) and is ultra-rare (not present in gnomAD). Proband AM1 (a case of non-syndromic bilateral microphthalmia and coloboma) was heterozygous for another missense variant, c.1511T>C; p.(Met505Thr). This variant, defined as likely pathogenic, is predicted to be deleterious (aggregated prediction score = 0.99) and is rare (gnomAD AF = 0.0004%). The probands’ affected brother (AM1-004) was also heterozygous for this variant. *HK3* encodes for hexokinase 3, an enzyme involved in glucose metabolism [47]. It is not associated with a known Mendelian phenotype in humans.

## 4. Discussion

This study presents genetic analysis by WES in 47 MAC affected individuals from 43 unrelated Israeli families of various ethnic backgrounds. Potentially causative variants were identified in 13/43 families (30%), broadly comparable to yields reported in the literature [6,9,10,11,12,13]. Utilization of other genetic diagnostic approaches, especially WGS, could have led to identification of non-coding and structural pathogenic variants in some of the unsolved families, including monoallelic cases. Nevertheless, it should be noted that recent studies reporting the results of short-read WGS in large MAC cohorts achieved modest diagnostic rates of 30% [6] and 18% [9]. In the latter study, WGS was used as a second-line analysis after targeted next-generation sequencing and/or CGH-array for 78% of the patients in the cohort. However, a similar diagnostic yield (15%) was observed for individuals without prior analysis [9]. Utilization of long-read WGS could lead to a preferable outcome, as this approach detects complex structural variants and variants located within repetitive regions, which might be missed by short-read WGS [48].

MAC in this cohort is highly genetically heterogeneous, with multiple genes and variant types identified with only limited recurrence, highlighting the need for comprehensive genomic evaluation. Overall, 10 genes were attributed to the MAC phenotype, with variants in *MFRP* occurring in three unrelated families. Of note, retinitis pigmentosa was identified with microphthalmia in all *MFRP*-mutant patients, a known phenotypic association for this gene [26,27]. MFRP is localized to the ciliary body and retinal pigment epithelium and is thought to play a role in ocular development [49]. *SMO* was associated with syndromic features only in a single patient, in whom dermatological and developmental findings were consistent with Curry–Jones syndrome, a multisystem disorder characterized by skin lesions, neurological and gastric malformations, as well as the MAC spectrum [35]. In family NGC12, three affected individuals had non-syndromic microphthalmia. This may be explained by the hypomorphic nature of the *SMO* variant segregating in this family, which might lead to a relatively mild phenotype. *SMO* encodes Smoothened, a key transducer of the Hedgehog (Hh) signaling pathway, and mutations in this gene result in constitutive pathway activation, leading to abnormal signaling during embryogenesis [35].

Additional ocular features were found in 19/47 patients (40%), comparable to the frequency reported in previous studies (44–60%) [3,6]. Likewise, consistent with prior reports, anterior segment abnormalities were the most prevalent findings in our cohort [3]. This pattern is well described in the MAC spectrum and likely reflects shared genetic and developmental pathways that govern early ocular morphogenesis [50,51]. Another common associated finding is PFV [3], which results from incomplete regression of the fetal hyaloid vascular system. In one case series, microphthalmia was present in 43% of PFV eyes and was significantly associated with a more severe PFV phenotype [52].

No significant correlation was found between laterality and the probability of genetic etiology. While the percentage of genetically solved patients was higher in bilateral versus unilateral cases (47% versus 26%, respectively), this difference was not statistically significant (*p* = 0.2057 using Fisher’s exact test).

Extra-ocular features were present in only 10/47 patients (21%), which is lower than the frequency reported in other MAC cohorts (33–50%) [3,6,7]. This difference may partly reflect the younger age of participants in our study (median age of five years old) compared with the median ages of 11–13 years in prior cohorts [3,6], as some systemic manifestations may emerge later in childhood. Even so, and consistent with the literature, neurological findings were the most prevalent extra-ocular features within our cohort. In four families with apparent extra-ocular involvement, WES analysis revealed that MAC is an isolated occurrence with other genetic findings explaining the non-MAC phenotypes.

Consanguinity was reported in 8/43 families (18.6%). The presence of consanguinity significantly elevated the probability of a genetic etiology, as genetic diagnosis was achieved in 6/8 consanguineous families (75%), compared to 7/35 non-consanguineous families (20%) (*p* = 0.0055 using Fisher’s exact test). Homozygous pathogenic variants in MAC-associated genes (*MFRP* and *TSPAN12*) were detected in four of the consanguineous families, confirming the expected AR inheritance mode. Surprisingly, in two of the consanguineous families the inheritance mode was changed to AD post-genetic analysis. Patient AA1-001, with non-syndromic microphthalmia, was heterozygous for a likely pathogenic missense variant in *SOX2*, inherited from her supposably unaffected mother. This variant was previously reported in a four-generation family, in which it segregated with a highly variable phenotype, ranging in severity from anophthalmia/microphthalmia to retinal tuft and refractive error [53]. Patient AW1-001, with non-syndromic microphthalmia, was heterozygous for an ultra-rare variant in the *SHH* gene. Heterozygous pathogenic variants in *SHH* are a known cause of non-syndromic microphthalmia/coloboma. Nevertheless, since the detected variant is defined as a VUS and was inherited from the unaffected father, the family was categorized as “possibly solved”.

Heterozygous variants in genes associated with dominantly inherited conditions, which were identified in isolated cases and inherited from supposably unaffected parents, were detected in three families: AA1 and AW1, which were discussed above, and NGC10. The latter family segregated a heterozygous ultra-rare variant with a deleterious prediction, in a gene (*SMO*) associated with a syndromic phenotype (Curry–Jones syndrome) compatible with the phenotype observed in the proband. While parents in each of these families may be truly unaffected due to incomplete penetrance, careful clinical examination will be required to assess the presence of sub-clinical manifestations. Nevertheless, these cases further support the involvement of additional genetic and/or environmental modifying factors which affect the final phenotypic outcome.

Pathogenic variants in *BEST1* are usually associated with inherited retinal degenerative diseases, including Best vitelliform macular dystrophy, adult-onset foveomacular vitelliform dystrophy, and AR bestrophinopathy. However, some rare variants have been associated with AD ocular development abnormalities similar to those of patient NGC14-001 [54]. Pathogenesis of *BEST1*-associated developmental defects involves altered protein localization and impaired chloride channel function, which disrupts the osmotic regulation and directional fluid transport essential for ocular expansion. This physiological breakdown, potentially exacerbated by increased retinal pigment epithelium cell apoptosis, restricts peripheral growth during early development, resulting in the microphthalmia–nanophthalmos spectrum [54,55].

In two patients, rare and potentially pathogenic variants were found in genes for which the association with MAC was reported only once before (*PTPN11* and *TP63*) [39]. While these findings might be coincidental, they may also represent novel genotype–phenotype associations that warrant further investigation.

Multiple rare and possibly pathogenic variants in genes not previously associated with MAC or with any phenotype in humans were identified in both solved and unsolved MAC patients. Each of these variants may contribute to the MAC phenotype, by itself or in combination with additional risk factors. Of specific interest are variants in *MCF2L2* and *HK3*, which were identified in more than one patient in this cohort. Establishing their involvement in MAC requires additional research, including identification of pathogenic variants in these genes in additional patients and functional analyses.

## 5. Conclusions

In conclusion, MAC is a clinically heterogeneous group of conditions, which can be caused by diverse etiologies, including both genetic and environmental factors. The current study is the first to present phenotypic and genotypic characterization of MAC in the Israeli population. The results further demonstrate the genetic heterogeneity of MAC, while supporting the involvement of complex inheritance and/or environmental factors in many of the cases. Further studies are required to reveal these underlying etiological factors, and to support the novel genotype–phenotype associations suggested here.

## Data Availability

The original contributions presented in this study are included in the article/Supplementary Materials. Further inquiries can be directed to the corresponding authors.

## Supplementary Materials

The following supporting information can be downloaded at: https://www.mdpi.com/article/10.3390/biom16081219/s1, Supplementary Materials S1: Sequences of splice products obtained in the minigene splice assay of the *SMO* c.1264+3G>C variant; Figure S1: Filtering strategy for variants obtained by exome sequencing of MAC patients; Table S1: Demographic and clinical data of study participants; Table S2: Segregation of identified variants in families; Table S3: Genes included in 1q21.1–q21.2 heterozygous deletion in patient TB1340/R2133; and Table S4: Variants of interest in exomes of MAC cases.

## Abbreviations

The following abbreviations are used in this manuscript:

AD	Autosomal dominant
AR	Autosomal recessive
CGH	Comparative genomic hybridization
CNVs	Copy number variants
gnomAD	Genome Aggregation Database
MAC	Microphthalmia, anophthalmia and ocular coloboma
PFV	Persistence of fetal vascularate
VUS	Variant of uncertain significance
WES	Whole exome sequencing
WGS	Whole genome sequencing
WT	Wild-type

## Appendix A

**Table A1:** Genetic findings in families with MAC.

Family	Clinical Diagnosis (Pre-Genetic Testing)	Clinical Diagnosis (Post-Genetic Testing) (MIM Number)	Inheritance Mode	Gene (GenBank Accession Number)	Pathogenic Variant/s	Aggregated Prediction Score (Franklin)	gnomAD Aggregated AF	ACMG Classification (ACMG Rules)	Ref
Solved families									
TB1517	Nanophthalmos and retinal dystrophy	Nanophthalmos 2 (609549)	AR	*MFRP* (NM_031433.4)	c.1125G>T; p.(Arg375Ser) hom	0.8 (deleterious)	0.0009%	VUS (PM2, PP3, PP4)	ClinVar: RCV001304447
TB1110	Nanophthalmos and retinal dystrophy	Nanophthalmos 2 (609549)	AR	*MFRP* (NM_031433.4)	c.1250del; p.(Thr417Argfs*61) hom	NA	-	Pathogenic (PVS1, PM2, PM3, PP5)	ClinVar: RCV001169878
TB533/ TB621	Microphthalmia and retinal dystrophy	Microphthalmia, isolated 5 (611040)	AR	*MFRP* (NM_031433.4)	Exon 1 deletion (start loss) hom	NA	-	Pathogenic (PVS1, PM2, PM3, PP1)	[56]
TB1340	Coloboma and cataract	Cataract 1, multiple types with ocular coloboma (116200)	AD	*GJA8* (NM_005267.5) (+31 genes) *	1q21.1-q21.2 deletion (Chr1: g.146,577,485-147,394,506) het	NA	NA	Pathogenic (1A, 2A, 3A, 4B)	[30]
AA1	Microphthalmia	Microphthalmia	AD	*SOX2* (NM_003106.4)	c.368A>G; p.(Asp123Gly) het	0.88 (deleterious)	-	Likely pathogenic (PS4, PM2, PP3, PP5)	[53]
NGC9	Microphthalmia, syndromic	Microphthalmia, syndromic 12 (615524)	AD (de novo)	*RARB* (NM_000965.5)	c.1196T>C; p.(Met399Thr) het	0.63 (uncertain)	-	Likely pathogenic (PM1, PM2, PM6, PP2, PP5)	-
NGC10	Anophthalmia, syndromic	Curry–Jones syndrome (601707)	AD	*SMO* (NM_005631.5)	c.1427A>G; p.(Asn476Ser) het	0.72 (deleterious)	-	VUS (PM2, PP3)	-
NGC34	Microphthalmia, retinal detachment, PFV, DD	Familial exudative vitreoretinopathy	AR	*TSPAN12 (NM_012338.4)*	c.542G>T; p.(Cys181Phe) hom	0.99 (deleterious)	-	Likely pathogenic (PM2, PS4, PP3)	[29]
Possibly solved families									
AW1	Microphthalmia	Microphthalmia/coloboma 5 (611638)	AD	*SHH* (NM_000193.4)	c.868G>T; p.(Gly290Cys) het	0.46 (uncertain)	-	VUS (PP2, PM2, PM5)	-
NGC4	Microphthalmia and anterior segment dysgenesis	Microphthalmia and anterior segment dysgenesis	AD (de novo)	*PTPN11* (NM_002834.5)	c.794G>A; p.(Arg265Gln) het	0.89 (deleterious)	0.0032%	Pathogenic (PS2, PS3, PS4, PM1, PM2, PM5, PP1, PP2, PP3, PP5)	[36]
NGC12	Microphthalmia	Microphthalmia	AD	*SMO* (NM_005631.5)	c.1264+3G>C het	0.56 (uncertain)	-	VUS (PM2, PP1)	-
NGC14	Microphthalmia, coloboma, microcornea	Microcornea, rod-cone dystrophy, cataract, and posterior staphyloma 2 (193220)	AD	*BEST1* (NM_004183.4)	c.1144G>A; p.(Glu382Lys) het	0.58 (uncertain)	0.0007%	VUS (PM2, PP2)	ClinVar: RCV002667004
NGC18	Anophthalmia, polydactyly, oligodactyly, ventricular septal defect	ADULT syndrome (103285)?	AD	*TP63* (NM_003722.5)	c.416C>T; p.(Ala139Val) het	0.6 (uncertain)	0.0014%	VUS (PM2, PP2)	-
	Polycystic kidney disease	Polycystic kidney disease 2 (173910)	AD	*PKD2* (NM_000297.4)	c.1094+1G>A het	0.8 (deleterious)	-	Pathogenic (PVS1, PS4, PM2, PP1)	[41]
Monoallelic families ^¶^									
NGC3	Microphthalmia	Microphthalmia, isolated 6 (613517)?	AR?	*PRSS56* (NM_001195129.2)	c.961del; p.(Val321Serfs*16) het	NA	0.0053%	Likely pathogenic (PVS1, PM2)	-
NGC16	Microphthalmia	Microphthalmia with limb anomalies (206920)?	AR?	*SMOC1* (NM_001034852.3)	c.832C>T; p.(Arg278Cys) het	0.64	0.0012%	VUS (PM2, PP5)	[57]
Unsolved families with genetic findings underlying non-MAC phenotypes									
ZKA1	Coloboma, microphthalmia	Coloboma, microphthalmia	isolate	-	-	-	-	-	-
	Hearing loss	Alport syndrome, X-linked (301050)	XLD	*COL4A5* (NM_033380.3)	c.4562C>G; p.(Thr1521Ser) hemi	0.87 (deleterious)	-	Likely pathogenic (PM2, PP2, PP3, PP5)	-
NGC11	Microphthalmia	Microphthalmia	isolate	-	-	-	-	-	-
	IUGR, hypothyroidism	Autoinflammation, immune dysregulation, and eosinophilia (AIIDE) (618999) (growth retardation and autoimmune thyroid disease included)	AD	*JAK1* (NM_002227.4)	c.911C>T; p.(Ser304Leu) het	0.2	0.0012%	VUS (PM2, PP2)	ClinVar: RCV002610623
NGC25	Microphthalmia	Microphthalmia	isolate	-	-	-	-	-	-
	Hearing loss	Deafness, autosomal recessive 111 (DFNB111)	AR	*MPZL2* (NM_005797.4)	c.72del; p.(Ile24Metfs*22) hom	NA	0.0769%	Pathogenic (PM, PVS1, PM2)	[44]
NGC29	Anophthalmia, microphthalmia, coloboma	Anophthalmia, microphthalmia, coloboma	isolate	*-*	-	-	-	-	-
	DD, epilepsy, ASD	Developmental delay, behavioral abnormalities, and neuropsychiatric disorders	AD	*ADGRL1* (NM_014921)	c.965T>C; p.(Val1322Ala)	0.52	0.0024%	VUS (PM2, PP2)	ClinVar: RCV004981543

* Complete list of genes included in the deleted region can be found on Table S3. ^¶^ Families with one heterozygous pathogenic allele in a gene compatible with autosomal recessive inheritance and with the observed phenotype, and a second heterozygous allele not found. A question mark (?) was added in a few cases where inheritance mode or diagnosis are possible but not definite. AD, autosomal dominant; AF, allele frequency; AR, autosomal recessive; ASD, autistic spectrum disorder; DD, developmental delay; het, heterozygous; hom, homozygous; IUGR, intrauterine growth retardation; MIM, Mendelian Inheritance in Man; NA, not applicable; PFV, persistent fetal vasculature; VUS, variant of unknown significance; XLD, X-linked dominant.
