# Supplementary material for: Characterization of Ocular Developmental Disorders in the Israeli Population: Genotype–Phenotype Correlations and Novel Candidate Genes
Source: Biomolecules. 2026 Aug 21;16(8):1219. doi: 10.3390/biom16081219 (PMC13510475; doi:10.3390/biom16081219)

Figure S1. Filtering strategy for variants obtained by exome sequencing of MAC patients

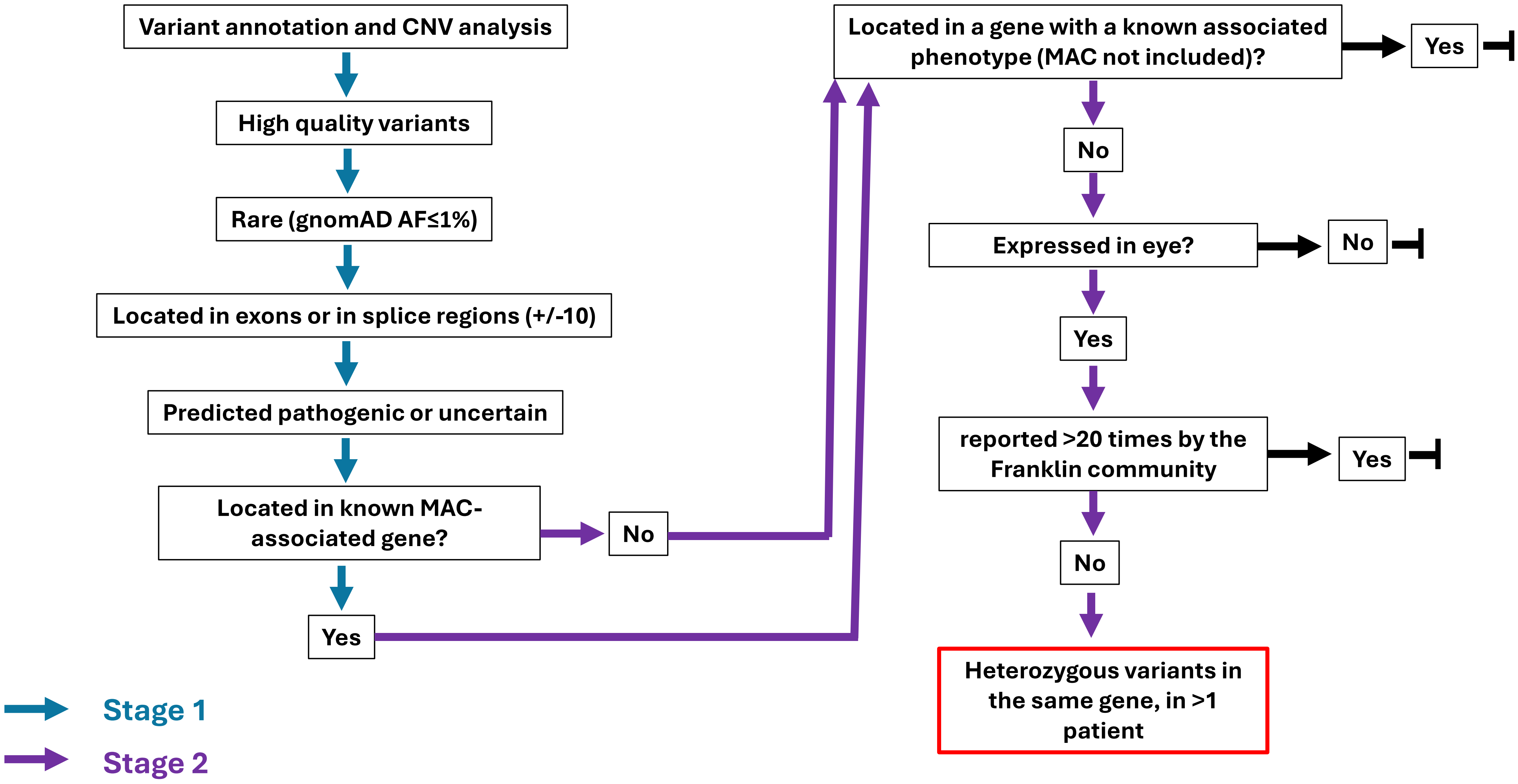

Supplement: Supplementary file 1 [file biomolecules-16-01219-s001.zip › Figure S1.pdf]
