# Supplementary material for: Characterization of Ocular Developmental Disorders in the Israeli Population: Genotype–Phenotype Correlations and Novel Candidate Genes
Source: Biomolecules. 2026 Aug 21;16(8):1219. doi: 10.3390/biom16081219 (PMC13510475; doi:10.3390/biom16081219)
Supplement: Supplementary file 1 [file biomolecules-16-01219-s001.zip › Supplementary Materials S1 minigene splice products sequences.pdf]

Supplementary Materials S1: Sequences of splice products obtained in the minigene splice assay of the *SMO* c.1264+3G>C variant

WT normal 2 (425bp)- normal splicing product
WT upper band 5 (791bp)- abnormal splicing product (intron 5+6 retention)
WT upper band 4 (464bp)- abnormal splicing product (partial intron 5 retention)
WT upper band 17 (700bp)- abnormal splicing product (intron 5 retention)

Var1 normal (425bp)- normal splicing product
Var1 upper band 10 (791bp)- abnormal splicing product (intron 5+6 retention)
Var1 12 (301bp)- abnormal splicing product (skipping of exon 6)
Var1 9 (150bp)- abnormal splicing product (uses splice sites within exons 5 and 6)
Var1 upper band 11 (452bp)- abnormal splicing product (partial intron 5 retention)

WT normal 2 (425bp)- normal splicing product

GCCATGGCGGCCGCGGGNATTCGATTGATATCGCATGGTCTCGTTGATCTTGCT
GGCAGCCTTCTCACTCAGCAGCCCGGGGTGGTTGCTCTTGATGGAGAACAGAGTC
ATGACTCCTCGGATGAGGAAGCAGCCTCCCACGATGAGCACCAGGCCGATTGGGG
CCAGCACGAAGCCCGCACGGTATCGGTAGTTCTTGTAGCCCACAAAACAA
ATGCCACTCACAGAGTCCCCATCCACCTGCGCCACAGCAAGGATTACCACAGTGG
GACAAAGGGGAGTGACCAGGTGAGCAGGTGGAAGTAGGAGGTCTTGCCCGAGAGA
GGCTGGTAGGTGGTGCCCAGGGCTTTGAAGGAAGTGTGCCAGGCATAGG
TGAGGACCACAAACCAAACCACACCAGCCATCAGGGCGTAGTACACGATGACAAA
GATGATGACGCAGGACAGAGTCTCATTGGAGCTCAATCACTAGTGAATTCGCGGCC
GCCTGCAGGTCGACCATATGGGAGAGCTCCCAACGCGTTGGATGCATAG
CTTGAGTATTCTATAGTGTCACCTAAATAGCTTGGCGTAATCATGGTCATAGCTGTTTCC
TGTGTGAAATTGTTATCCGCTCACAATTCCACACAACATACGAGCCGGAAGCATAAAG
TGTAAAGCCTGGGGTGCCTAATGAGTGAGCTAACTCACATTA
ATTGCGTTGCGCTCACTGCCCGCTTTCCAGTCGGGAAACCTGTCGTGCCAGCTGCA
TTAATGAATCGGCCAACGCGCGGGGAGAGGCGGTTTGCGTATTGGGCGCTCTTCC
GCTTCCTCGCTCACTGACTCGCTGCGCTCGGTCGTTCGGCTGCGGCGAG
CGGTATCAGCTCACTCAAAGGCGGTAATACGGTTATCCACAGAATCNGGGGATAAC
GCAGGAAAGAACATGTGAGCAAAAGGCCAGCAAAGGCCAGGAACCGTAAAAAGG
CCGCGTTGCTGGCGTTTTTCCAT

WT upper band 5 (791bp)- abnormal splicing product (intron 5+6 retention)

TGGCGGCCGCGGGAATTCGATTGAGCTCCAATGAGACTCTGTCCTG
CGTCATCATCTTTGTCATCGTGTACTACGCCCTGATGGCTGGTGTGGTTTGGTTTGTGG
TCCTCACCTATGCCTGGCACACTTCCTTCAAAGCCCTGGGCACCACCTACCAGCCT
CTCTCGGGCAAGACCTCCTACTTCCACCTGCTCACCTGGTCACTC
CCCTTTGTCCTCACTGTGGCAATCCTTGCTGTGGCGCAGGTATAGTGACTGGTAGGA
ACGGGAGACCTGGATGGGGTGAGTTTGAGGGAGGGGGCCAGTAACCCACCTTCTG
TCCCACCCCTTCCTGCTGCAGGTGGATGGGGACTCTGTGAGTGGCATT
TGTTTTGTGGGCTACAAGAACTACCGATACCGTGCGGGCTTCGTGCTGGCCCCAAT
CGGCCTGGTGCTTATCGTGGGAGGCTACTTCCTCATCCGAGGTGAGTGAAGACCAG
GCTAGGACCAGTTGGGCAACAAAATGTACTGGGCACTTGCTGCCAGTA
CTGGGAGCTGCCAGCACGGCTGCCCCCATGCTGAAACCCCAGCTAGCTCCTAGG
ATCCCCATTTCTCCAGTGTCTCTAGCTCCCCAGGTTTTCATTTAGCACAGGGGTAATC
AGACTTGGGACTCCAGAGCCTTANGACCCTCCTCCCACTCACCCATCC
TTCCCAGCGGGGCAGCCTCACCCCTGCTAATGTCTGAGGTCCCCCTTCTGTTCANG
AGTCATGACTCTGTTCTCCATCAAGAGCAACCACCCCGGGCTGCTGAGTGAGAAGG
CTGCCAGCAAGATCAACGAGACCATGCGATATCAATCACTAGTGAATT
CGCGGGCCGCCTGCAGGTCGACCATATGGGAGAGCTNCCAACGCGTTGGATGCA
TAGCTTGAGTATTCTATAGTGTCACCTAAATAGCTTGGGCGTAATCATGG

WT upper band 4 (464bp)- abnormal splicing product (partial intron 5 retention)

ATGGCGGCCGCGGGNATTCGATTGAGCTCCAATGAGACTCTGTCCTGCG
TCATCATCTTTGTCATCGTGTACTACGCCCTGATGGCTGGTGTGGTTTGGTTTGTGGTC
CTCACCTATGCCTGGCACACTTCCTTCAAAGCCCTGGGCACCACCTACCAGCCTCT
CTCGGGCAAGACCTCCTACTTCCACCTGCTCACCTGGTCACTCCC
CTTTGTCCTCACTGTGGCAATCCTTGCTGTGGCGCAGGTATAGTGACTGGTAGGAAC
GGGAGACCTGGATGGGGTGAGTTCGAGGGAGGGGGCCAGTAACCCACCTTCTGTC
CCACCCCTTCCTGCTGCAGGTGGATGGGGACCCTGTGAGTGGCATTTG
TTTTGTGGGCTACAAGAACTACCGATACCGTGCGGGCTTCGTGCTGGCCCCAATCG
GCCTGGTGCTCATCGTGGGAGGCTACTTCCTCATCCGAGGAGTCATGACTCTGTTCT
CCATCAAGAGCAACCACCCCGGGCTGCTGAGTGAGAAGGCTGCCAGC
AAGATCAACGAGACCATGCGATATCAATCACTAGTGAATTCGCGGCCGCCTGCAGG
TCGACCATATGGGAGAGCTCCCAACGCGTTGGATGCATAGCTTGAGTATTCTATAGTG
TCACCTAAATAGCTTGGCGTAATCATGGTCATAGCTGTTTCCTGTG
TGAAATTGTTATCCGCTCACAATTCCACACAACATACGAGCCGGAAGCATAAAGTGTA
AAGCCTGGGGTGCCTAATGAGTGAGCTAACTCACATTAATTGCGTTGCGCTCACTGC
CCGCTTTCCAGTCGGGAAACCTGTCGTGCCAGCTGCATTAATGAA
TCGGGCCAACGCGCGGGGAGAGGCGGTTTGCGTATTGGGCGCTCTTCCGCTTCCT
CGCTCACTGACTCGCTGCGCTCGGTCGTTCGGCTGCGGCGAGCGGTATCAGCTCA
CTCAAAAGC

WT upper band 17 (700bp)- abnormal splicing product (intron 5 retention)

ATTCGATTGATATCGCATGGTCTCGTTGATCTTGCTGG
CAGCCTTCTCACTCAGCAGCCCGGGGTGGTTGCTCTTGATGGAGAACAGAGTCATG
ACTCCTGAACAGAAGGGGGACCTCAGACATTAGCAGGGGTGAGGCTGCCCTGCTG
GGAAGGATGGGTGAGTGGGAGGAGGGTCCTAAGGCTCTGGAGTCCCAAG
TCTGATTACCCCTGTGCTAAATGAAAACCTGGGGAGCTAGAGACACTGGAGAAATGG
GGATCCTAGGAGCTAGCTGGGGTTTCAGCATGGGGGCAGCCGTGCTGGCAGCTCC
CAGTACTGGCAGCAAGTGCCCAGTACATTTTGTTGCCCAACTGGTCCT
AGCCTGGTCTTCACTCACCTCGGATGAGGAAGTAGCCTCCCACGATGAGCACCAG
GCCGACTGGGGCCAGCACGAAGCCCGCACGGTATCGGTAGTTCTTGTAGCCCACA
AAACAAATGCCACTCACAGAGTCCCCATCCACCTGCGCCACAGCAGGGAT
TGCCACAGTGAGGACAAAGGGGAGTGACCAGGTGAGCAGGTGGAAGTAGGAGGTC
TTGCCCGAGAGAGGCTGGTAGGTGGTGCCCAGGGCTTTGAAGGAAGTGTGCCAGG
CATAGGTGAGGACCACAAACCAAACCACACCAGCCATCAGGGCGTAGTAC
ACGATGACAAAGATGATGACGCANGACAGAGTCTCATTGGAGCTCAATCACTAGTGA
ATTCGCGGNCGCCTGCAGGTCGACCATATGGGAGAGCTCCCNACGCGTTGGATGC
ATAGCTTGAGTATTCTATAGTGTCACCTAAATAGCTTGGCGTAATCAT
GGTCATAGCTGTTTCCTGTGTGAAATTGTTATCCGCTCNCATTTCCACACAACATACGA
GCCGGAAGCNNNAAAGTGNAA
AGCCTGGGGGNGCCTAATGAGTGAGCTTAC

Var1 normal (425bp)- normal splicing product

ATGGCGGCCGCGGGAATTCGATTGATATCGCATGGTCTCGTTGAT
CTTGCTGGCAGCCTTCTCACTCAGCAGCCCGGGGTGGTTGCTCTTGATGGAGAACA
GAGTCATGACTCCTCGGATGAGGAAGTAGCCTCCCACGATGAGCACCAGGCCGATT
GGGGCCAGCACGAAGCCCGCACGGTATCGGTAGTTCTTGTAGCCCACA
AAACAAATGCCACTCACAGAGTCCCCATCCACCTGCGCCACAGCAAGGATTGCCA
CAGTGAGGACAAAGGGGAGTGACCAGGTGAGCAGGTGGAAGTAGGAGGTCTTGCC
CGAGAGAGGCTGGTAGGTGGTGCCCAGGGCTTTGAAGGAAGTGTGCCAGG
CATAGGTGAGGACCACAAACCAAACCACACCAGCCATCAGGGCGTAGTACACGAT
GACAAAGATGATGACGCAGGACAGAGTCTCATTGGAGCTCAATCACTAGTGAATTCG
CGGCCGCCTGCAGGTCGACCATATGGGAGAGCTCCCAACGCGTTGGAT
GCATAGCTTGAGTATTCTATAGTGTCACCTAAATAGCTTGGCGTAATCATGGTCATAGCT
GTTTCCTGTGTGAAATTGTTATCCGCTCACAATTCCACACAACATACGAGCCGGAAGC
ATAAAGTGTAAAGCCTGGGGTGCCTAATGAGTGAGCTAACTC
ACATTAATTGCGTTGCGCTCACTGCCCGCTTTCCAGTCGGGAAACCTGTCGTGCCA
GCTGCATTAATGAATCGGCCAACGCGCGGGGAGAGGCGGTTTGCGTATTGGGCGC
TCTTCCGCTTCCTCGCTCACTGACTCGCTGCGCTCGGTCGTTCGGCTGC
GGCGAGCGGTATCAGCTCACTCAAAGGCGGTAATACGGTTATCCACAGAATCAGGG
GATAACGCAGGAAAGAACATGTGAGCAAAAGGCCAGCAAAAGGCAGGAACCGTAA
AAAGGCCGCGTTGCTGGCGNTTTTNCATAA

Var1 upper band 10 (791bp)- abnormal splicing product (intron 5+6 retention)

CGCCATGGCGGCCGCGGGAATTCGATTGAGCTCCAATGAGACTCTGTCCTGCG
TCACCATCTTTGTCATCGTGTACTACGCCCTGATGGCTGGTGTGGTTTGGTTTGTGGTC
CTCACCTATGCCTGGCACACTTCCTTCAAAGCCCTGGGCACCACCTACCAGCCTCT
CTCGGGCAAGACCTCCTACTTCCACCTGCTCACCTGGTCACTCCC
CTTTGTCCTCACTGTGGCAATCCTTGCTGTGGCGCAGGTATAGTGACTGGTAGGAAC
GGGAGACCTGGGTGGGGTGAGTTTGAGGGAGGGGGCCAGTAACCCACCTTCTGTC
CCACCCCTTCCTGCTGCAGGTGGATGGGGACTCTGTGAGTGGCATTTG
TTTTGTGGGCTACAAGAACTACCGATACCGTGCGGGCTTCGTGCTGGCCCCAATCG
GCCTGGTGCTCACCGTGGGAGGCTACTTCCTCATCCGAGGTCAGTGAAGACCAGG
CCAGGACCAGTTGGGTAACAAAATGTACTGGGCACTTGCTGCCAGTACT
GGGAGCTGCCAGCACGGCTGCCCCCATGCTGAAACCCCAGCTAGCTCCTAGGAT
CCCCATTTCTCCAGTGTCTCTAGCTCCCCAGGTTTTCATTTAGCACAGGGGTAATCAG
ACTTGGGACTCCAGAGCCTTAGGACCCTCCTCCCACTCACCCATCCTT
CCCAGCAGGGCAGCCTCACCCCTGCTAATGTCTGAGGTCCCCCTTCTGTTCAGGA
GTCATGACTCTGTTCTCCATCAAGAGCAACCACCCCGGGCTGCTGAGTGAGAAGGC
TGCCAGCAAGATCAACGAGACCATGCGATATCAATCACTAGTGAATTCG
CGGCCGCCTGCAGGTCGACCATATGGGAGAGCTCCCAACGCGTTGGATGCATAGC
TTGAGTATTCTATAGTGTCACCTAAATAGCTTGGCGTAATCATGGGTCATAGCTGTTTCC
TGTGTGAAATTGTTATCCGCTCACAATTCCNNNCAACATACGAGC
CGGAAGCATAAAGTGTAAAGCCTGGGGNGCCCTAATGAGTGAGCTAACTCACCNTT
AATTGCGTTTGCNCNTCACTGCCC
GCTTTCCANTCGGAAAACCT

Var1 12 (301bp)- abnormal splicing product (skipping of exon 6)

TGGCGGCCGCGGGAATTCGATTGATATCGCATGGTCTCGTTGATCTTGCT
GGCAGCCTTCTCACTCAGCAGCCCGGGGTGGTTGCTCTTGATGGAGAACAGAGTC
ATGACTCCTGCGCCACAGCAAGGATTGCCACAGTGAGGACAAAGGGGAGTGACCA
GGTGAGCAGGTGGAAGTAGGAGGTCTTGCCCGAGAGAGGCTGGTAGGTGG
TGCCCAGGGCTTTGAAGGAAGTGTGCCAGGCATAGGTGAGGACCACAAACCAAAC
CACACCAGCCATCAGGGCGTAGTACACGATGACAAAGATGATGACGCAGGACAGA
GTCTCATTGGAGCTCAATCACTAGTGAATTCGCGGCCGCCTGCAGGTCGA
CCATATGGGAGAGCTCCCAACGCGTTGGATGCATAGCTTGAGTATTCTATAGTGTCAC
CTAAATAGCTTGGCGTAATCATGGTCATAGCTGTTTCCTGTGTGAAATTGTTATCCGCTC
ACAATTCCACACAACATACGAGCCGGAAGCATAAAGTGTAAA
GCCTGGGGTGCCTAATGAGTGAGCTAACTCACATTAATTGCGTTGCGCTCACTGCCC
GCTTTCCAGTCGGGAAACCTGTCGTGCCAGCTGCATTAATGAATCGGCCAACGCGC
GGGGAGAGGCGGTTTGCGTATTGGGCGCTCTTCCGCTTCCTCGCTCA
CTGACTCGCTGCGCTCGGTCGTTCGGCTGCGGCGAGCGGTATCAGCTCACTCAAA
GGCGGTAATACGGTTATCCACAGAATCAGGGGATAACGCAGGAAAGAACATGTGAG
CAAAAGGCCAGCAAAAGGCCAGGAACCGTAAAAAGGCCGCGTTGCTGGC
GTTTTTCCATNNCTCCGCCCCCCTGACGAGCATCACAAAAATCGACGCTCAAGTCA
GAGGTGGCGAAACCCGACAGGACTATAAAGATACCAGGCGTTTCCCCCTGGAAAG
CTCCCTCGNGCGCTCTCCTGTTCCGACCCTGCCGCTTACCGGGATACCT
GTCCGCCTTTCTCCCTTTCGGGGAAGCG

Var1 9 (150bp)- abnormal splicing product (uses splice sites within exons 5 and 6)

TCCCGGCCGCCATGGCGGCCGCGGGAATTCGATTGAGCTCCAATGAGACTCTGTC
CTGCGTCATCATCTTTGTCATCGTGGGAGGCTACTTCCTCATCCGAGGAGTCATGACT
CTGTTCTCCATCAAGAGCAACCACCCCGGGCTGCTGAGTGAGAAGGCTGCCAGCA
AGATCAACGAGACCATGCGATATCAATCACTAGTGAATTCGCGGCCGCC
TGCAGGTCGACCATATGGGAGAGCTCCCAACGCGTTGGATGCATAGCTTGAGTATTC
TATAGTGTCACCTAAATAGCTTGGCGTAATCATGGTCATAGCTGTTTCCTGTGTGAAATT
GTTATCCGCTCACAATTCCACACAACATACGAGCCGGAAGCAT
AAAGTGTAAAGCCTGGGGTGCCTAATGAGTGAGCTAACTCACATTAATTGCGTTGCG
CTCACTGCCCGCTTTCCAGTCGGGAAACCTGTCGTGCCAGCTGCATTAATGAATCG
GCCAACGCGCGGGGAGAGGCGGTTTGCGTATTGGGCGCTCTTCCGCT
TCCTCGCTCACTGACTCGCTGCGCTCGGTCGTTCGGCTGCGGCGAGCGGTATCAG
CTCACTCAAAGGCGGTAATACGGTTATCCACAGAATCAGGGGATAACGCAGGAAAG
AACATGTGAGCAAAAGGCCAGCAAAAGGCCAGGAACCGTAAAAAGGCCG
CGTTGCTGGCGTTTTTCCATAGGCTCCGCCCCCCTGACGAGCATCACAAAAATCGA
CGCTCAAGTCAGAGGTGGCGAAACCCGACAGGACTATAAAGATACCAGGCGTTTCC
CCCTGGAAGCTCCCTCGTGCGCTCTCCTGTTCCGACCCTGCCGCTTAC
CGGATACCTGTCCGCCTTTCTCCCTTCGGGAAGCGTGGCGCTTTCTCATAGCTCAC
GCTGTAGGTATCTCAGTTCGGTGTAGGTCGTTCGCTCCAAGCTGGGCTGTGTGCACG
AACCCCCCGTTCAGCCCGACCGCTGCGCCTTATCCGGGTAACTATCG
TCTTGAGTCCAACCCGGTAAGACAC

Var1 upper band 11 (452bp)- abnormal splicing product (partial intron 5 retention)

CCGCCATGGCGGCCGCGGGAATTCGATTGATATCGCATGGTCTCGTTGATCTTGC
TGGCAGCCTTCTCACTCAGCAGCCCGGGGTGGTTGCTCTTGATGGAGAACAGAGTC
ATGACTCAGAGTCCCCATCCACCTGCGCCACAGCAAGGATTGCCACAGTGAGGAC
AAAGGGGAGTGACCAGGTGAGCAGGTGGAAGTAGGAGGTCTTGCCCGAG
AGAGGCCGGTAGGTGGTGCCCAGGGCTTTGAAGGAAGTGTGCCAGGCATAGGTGA
GGACCACAAACCAAACCACACCAGCCATCAGGGCGTAGTACACGATGACAAAGAT
GATGACGCAGGACAGAGTCTCATTGGAGCTCAATCACTAGTGAATTCGCG
GCCGCCTGCAGGTCGACCATATGGGAGAGCTCCCAACGCGTTGGATGCATAGCTT
GAGTATTCTATAGTGTCACCTAAATAGCTTGGCGTAATCATGGTCATAGCTGTTTCCTGT
GTGAAATTGTTATCCGCTCACAATTCCACACAACATACGAGCCGG
AAGCATAAAGTGTAAAGCCTGGGGTGCCTAATGAGTGAGCTAACTCACATTAATTGCG
TTGCGCTCACTGCCCGCTTTCCAGTCGGGAAACCTGTCGTGCCAGCTGCATTAATG
AATCGGCCAACGCGCGGGGAGAGGCGGTTTGCGTATTGGGCGCTCT
TCCGCTTCCTCGCTCACTGACTCGCTGCGCTCGGTCGTTCGGCTGCGGCGAGCG
GTATCAGCTCACTCAAAGGCGGTAATACGGTTATCCACAGAATCAGGGGATAACGCA
GGAAAGAACATGTGAGCAAAAGGCCAGCAAAAGGCCAGGAACCGTAAA
AAGGCCGCGTTGCTGGCGTTTTTCCATAGGCTCCGCCCCCCTGACGAGCATCACA
AAAATCGACGCTCAAGTCAGAGGNGNCGAAACCCGACAGGACTATAAAGATACCA
GGCGTTTCCCCCTGGAAGCTCCCTCGTGCGCTCTCCTNNTTCCGACCCTGC
GCTTACCGGATACCTGTC
