# Supplementary material for: Characterization of Ocular Developmental Disorders in the Israeli Population: Genotype–Phenotype Correlations and Novel Candidate Genes
Source: Biomolecules. 2026 Aug 21;16(8):1219. doi: 10.3390/biom16081219 (PMC13510475; doi:10.3390/biom16081219)
Supplement: Supplementary file 1 [file biomolecules-16-01219-s001.zip › Supplementary Table S1 Demographic revised YR-TBY.pdf]

Supplementary Table S1. Demographic and clinical data of study participants

**Family**	**Ethnicity**	**Consanguinity**	**Patient**	**Sex**	**MAC type**	**Laterality**	**Additional ocular phenotypes**	**Extra-ocular phenotypes**	**Inheritance mode (pre-genetic testing)**	**Inheritance mode (post-genetic testing)**
**Genetically solved families**										
TB1517	MA	Yes	R2366	F	microphthalmia	BE	RP	-	AR	AR
TB1110	OJ	Yes	R1844	M	microphthalmia	BE	cataract, glaucoma, RP	-	AR	AR
TB533/ TB621	MA	Yes	R1005	M	microphthalmia	BE	RP	-	AR	AR
			R1144	M	microphthalmia	BE	RP	-		
TB1340	MJ	No	R2133	M	coloboma	BE	cataract, exophthalmia (RE)**,** high myopia	-	**isolate**	**AD**
AA1	MA	Yes	001	F	microphthalmia	RE	-	-	**AR**	**AD**
NGC9	MA	No	001	F	microphthalmia	LE	-	Developmental delay, dysmorphism, cardiac, pulmonary, skeletal abnormalities	**isolate**	**AD (de novo)**
NGC10	MJ	No	001	F	anophthalmia	RE	-	Developmental delay, hypopigmented skin lesions	**isolate**	**AD**

NGC34	MA	Yes	001	M	microphthalmia	BE	retinal detachment, PFV	Developmental delay	**isolate**	**AR**
**Possibly solved families**										
AW1	MA	Yes	001	M	microphthalmia	LE	PFV	-	**AR**	**AD**
NGC4	ASH	No	001	M	microphthalmia	RE	anterior segment dysgenesis (LE)	-	**isolate**	**AD (de novo)**
NGC12	OJ	No	001	F	microphthalmia	RE	-	-	AD	AD
			002	F	microphthalmia	LE	-	-		
			003	F	microphthalmia	RE	-	-		
NGC14	OJ	No	001	F	microphthalmia, coloboma	LE	microcornea	-	AD (partial penetrance)	AD (partial penetrance)
NGC18	MA	No	001	F	anophthalmia	BE	-	polydactyly, oligodactyly, ventricular septal defect, kidney dysfunction	AD?	AD
**Unsolved families**										
TB755	MA	No	R1347	F	coloboma	LE	RP	Developmental delay	isolate	isolate
TB988	MJ	No	R1675	M	coloboma	BE	-	-	AD?	AD?
AM1	OJ	No	001	M	microphthalmia, coloboma	BE	-	-	AR? XLR? AD (partial penetrance)?	AR? XLR? AD (partial penetrance)?
			004	M	microphthalmia, coloboma	BE	-	-		
BS1	MJ	No	001	F	coloboma	RE	-	-	isolate	isolate

WR1	MA	Yes	001	F	microphthalmia	BE	congenital cataract (LE)	-	AR	AR
ZK1	MA	No	001	F	microphthalmia	LE	microcornea, hypermetropia, lymphangioma	-	isolate	isolate
ZKA1	ASH	No	001	M	coloboma<br>microphthalmia	BE<br>RE	-	hearing loss	isolate	isolate
NGC1	MA	Yes	001	F	anophthalmia	BE	-	-	AR	AR
NGC2	CA	No	001	M	coloboma	BE	-	-	isolate	isolate
NGC3	MJ	No	001	F	microphthalmia	RE	-	-	isolate	isolate
NGC5	OJ	No	001	M	microphthalmia	RE	cataract, PFV, retinal detachment	-	isolate	isolate
NGC6	ASH	No	001	F	microphthalmia, coloboma	LE	-	-	isolate	isolate
NGC7	MJ	No	001	F	microphthalmia	RE	retinal detachment, PFV	-	isolate	isolate
NGC8	ASH	No	001	F	microphthalmia, coloboma	LE	-	-	isolate	isolate
NGC11	ASH	No	001	M	microphthalmia	LE	-	hypothyroidism, intrauterine growth retardation	isolate	isolate
NGC13	MJ	No	001	F	microphthalmia	RE	PFV	-	isolate	isolate
NGC16	ASH	No	001	M	microphthalmia	LE	-	-	isolate	isolate
NGC17	ASH	No	001	F	microphthalmia, coloboma	LE	cataract	-	isolate	isolate

NGC19	MJ	No	001	F	microphthalmia	RE	-	-	AD (partial penetrance)	AD (partial penetrance)
NGC20	MJ	No	001	F	microphthalmia	RE	Morning glory disc	-	isolate	isolate
NGC21	OJ	No	001	F	microphthalmia	LE	-	-	isolate	isolate
NGC22	OJ	No	001	F	microphthalmia	LE	-	-	AD	AD
NGC24	OJ	No	001	F	microphthalmia	RE	-	-	isolate	isolate
NGC25	ASH	No	001	M	microphthalmia	RE	cataract	Developmental delay, hearing loss	isolate	isolate
NGC26	OJ	No	001	F	microphthalmia	RE	Peters anomaly, retinal detachment	-	isolate	isolate
NGC27	ASH	No	001	M	microphthalmia	LE	-	-	isolate	isolate
NGC29	MJ	No	001	M	anophthalmia<br>microphthalmia, coloboma	RE<br>LE	-	Developmental delay, epilepsy	isolate	isolate
NGC30	MJ	No	001	F	microphthalmia, coloboma	BE	nystagmus, PFV	-	isolate	isolate
NGC31	MJ	No	001	M	microphthalmia	LE	-	Crohn's disease, Non-Hodgkin lymphoma	isolate	isolate
NGC32	ASH	No	001	M	microphthalmia	RE	cataract, PFV	-	isolate	isolate

AD, autosomal dominant; AR, autosomal recessive; F, female; LE, left eye; M, male; PFV, persistent fetal vasculature; RE, right eye; RP, retinitis pigmentosa; XLR, X-linked recessive
