# Supplementary material for: Characterization of Ocular Developmental Disorders in the Israeli Population: Genotype–Phenotype Correlations and Novel Candidate Genes
Source: Biomolecules. 2026 Aug 21;16(8):1219. doi: 10.3390/biom16081219 (PMC13510475; doi:10.3390/biom16081219)
Supplement: Supplementary file 1 [file biomolecules-16-01219-s001.zip › Supplementary Table S2 segragation revised V3.pdf]

Supplementary table S2. Segregation of identified variants in families

Family	Gene	variant	Proband		Mother		Father		Additional family members		
			Phenotype	Genotype	Phenotype	Genotype	Phenotype	Genotype	Relative	Phenotype	Genotype
**Solved families**											
TB533/ TB621	*MFRP*	Exon 1 deletion	affected	v/v	unaffected	NT	unaffected	NT	Brother	affected	v/v
AA1	*SOX2*	c.368A>G; p.(Asp123Gly)	affected	v/+	unaffected	v/+	unaffected	+/+	-	-	-
NGC9	*RARB*	c.1196T>C; p.(Met399Thr)	affected	v/+ (de novo)	unaffected	+/+	unaffected	+/+	-	-	-
NGC10	*SMO*	c.1427A>G; p.(Asn476Ser)	affected	v/+	unaffected	NT	unaffected	v/+	-	-	-
**Possibly solved families**											
AW1	*SHH*	c.868G>T; p.(Gly290Cys)	affected	v/+	unaffected	NT	unaffected	v/+	-	-	
NGC4	*PTPN1 1*	c.794G>A; p.(Arg265Gln)	affected	v/+ (de novo)	unaffected	+/+	unaffected	+/+	-	-	
NGC12	*SMO*	c.1264+3G>C	affected	v/+	affected	v/+	unaffected	NT	Maternal grandmother	affected	v/+
NGC14	*BEST1*	c.1144G>A; p.(Glu382Lys)	affected	v/+	unaffected	+/+	unaffected	v/+	Paternal grandmother	affected	NT
NGC18	*TP63*	c.416C>T; p.(Ala139Val)	affected	v/+	affected	v/+	unaffected	+/+	-	-	-
	PKD2	c.1094+1G>A	affected	v/+	affected	v/+	unaffected	+/+	-	-	-
**Monoallelic families**											
NGC16	*SMOC 1*	c.832C>T; p.(Arg278Cys)	affected	v/+	unaffected	v/+	unaffected	+/+	-	-	-

NT, not tested; v, variant; +, wild-type allele
