# Supplementary material for: Characterization of Ocular Developmental Disorders in the Israeli Population: Genotype–Phenotype Correlations and Novel Candidate Genes
Source: Biomolecules. 2026 Aug 21;16(8):1219. doi: 10.3390/biom16081219 (PMC13510475; doi:10.3390/biom16081219)
Supplement: Supplementary file 1 [file biomolecules-16-01219-s001.zip › Supplementary Table S3 genes in deletion.pdf]

Supplementary Table S3. Genes included in 1q21.1-q21.2 heterozygous deletion in patient TB1340/R2133

Gene	Known phenotype (MIM#)	Inheritance pattern
**GJA8**	Cataract 1, multiple types (116200)	AD
GJA5	Atrial standstill 1 (108770)	digenic (GJA5/SCN5A)
	Atrial fibrillation, familial, 11 (614049)	AD
CHD1L	-	
ACP6	-	
MIR6077	-	
RNVU1-8	-	
PDZK1P1	-	
LINC00624	-	
NBPF8	-	
NBPF25P	-	
LINC01138	-	
RNVU1-3	-	
PPIAL4G	-	
PDIA3P1	-	
LOC728989	-	
BCL9	-	
NBPF26	-	
GPR89B	-	
NBPF13P	-	
PPIAL4D	-	
NBPF11	-	
RNVU1-14	-	
RNVU1-7	-	
PRKAB2	-	
PDE4DIPP6	-	
PDE4DIPP1	-	
FMO5	-	
LINC02805	-	
RNVU1-1	-	
LOC101927468	-	
MIR5087	-	
NBPF14	-	
